# Comparison of the Diagnostic Consistency between Delayed Radiographs taken Two Hours and Twenty-four Hours Post Hysterosalpingography using Ultra-Fluid Lipiodol-based Contrast Medium

**DOI:** 10.2174/0115734056258980231112082655

**Published:** 2025-09-18

**Authors:** Yitang Wang

**Affiliations:** 1 Department of Hemangioma and Intervention, Dalian Women and Children’s Medical Group, Dalian, China; £ Present Address: Shenyang Medical College Affiliated 242 Hospital First Interventional Department, China

**Keywords:** Hysterosalpingography, Ultra-fluid lipiodol, Delayed radiographs, Fallopian tube patency, Uterine and pelvic cavity morphology, Radiographs

## Abstract

**Background:**

Hysthyosalpingography (HSG) is commonly used to diagnose fallopian tubal disease. At the same time, a 24-hour interval is needed for taking delayed radiographs post-HSG using an oil-based contrast medium, which is inconvenient.

**Objective:**

This study used an Ultra-Fluid Lipiodol-based contrast medium to compare the diagnostic consistency between delayed radiographs taken 2 hours and 24 hours post-HSG.

**Methods:**

In total, 78 patients who received HSG examinations using ultrafluid lipiodol were enrolled in this cohort study. Then, after 2 hours and 24 hours, delayed radiographs were taken, which were subsequently randomized and assigned to two folders and read by investigators to assess the patency of the fallopian tubes, uterine morphology, and pelvic cavity morphology.

**Results:**

The delayed radiographs that were taken 2 hours and 24 hours post-HSG revealed substantial agreement in the diagnosis of fallopian tube patency (with a Gwet’s AC1 value of 0.624) and almost perfect agreement in determining uterine morphology (with a Gwet’s AC1 value of 0.943) and pelvic cavity morphology (with a Gwet’s AC1 value of 0.876). Twenty-nine (37.2%) and 3 (3.8%) patients experienced mild and moderate pain, respectively, and 3 (3.8%) patients suffered countercurrent blood flow during the HSG. After HSG, only 9 (11.5%) patients were exposed to mild pain. Vaginal bleeding did not occur either during or after HSG.

**Conclusion:**

Taking delayed radiographs 2 hours post-HSG using Ultra-Fluid Lipiodol exhibits high consistency in evaluating tubal patency and uterine and pelvic cavity morphology compared with the traditional 24-hour scheme.

## 1 INTRODUCTION

Fallopian tubal disease is one of the most common gynecological diseases in women and might lead to a series of concomitant diseases, such as infertility [1-5]. Hysterosalpingography (HSG) is widely used in the diagnosis of fallopian tubal disease, among which oil-based and water-based contrast mediums are widely applied; the former indicates better visualization and lower liquidity than the latter, which brings a higher accuracy for the diagnosis of fallopian tubal disease [6-10]. However, HSG with traditional oil-based contrast mediums requires 24-hour delayed radiographs to reveal fallopian tubal disease (such as hydrosalpinx), which is inconvenient for apprehensive patients waiting for HSG findings [11-13].

Ultraffluid lipiodol, or ethiodized poppy seed oil, is an organoiodine compound isolated from poppy seeds and iodine, exhibiting an antispasmodic effect (during HSG). In addition, Ultra-Fluid Lipiodol discloses an intermediate viscosity between the traditional oil contrast medium and the water-based contrast medium [14, 15], which allows a faster speed of intrauterine dispersion and implies a shorter waiting time for taking delayed radiographs. Some researchers have tried to shorten the time for delayed radiographs during HSG by using Ultra-Fluid Lipiodol [16]. For instance, one preliminary study explored taking delayed radiographs within 6 hours after HSG using Ultra-Fluid Lipiodol. This shows that the diagnosis finding in this group has a high kappa value compared with those taking delayed radiographs 24 hours after HSG [16]. However, with the accelerating pace of life, a 6-hour waiting period is still too long for contemporary youth, bringing unnecessary costs and restlessness.

Hence, the current study enrolled 78 patients who received HSG examinations using Ultra-Fluid Lipiodol as the contrast medium and obtained two delayed radiographs (at 2 hours and 24 hours post-HSG, separately), aiming to evaluate the feasibility of diagnosing tubal patency and uterine and pelvic cavity morphology by using 2-hour delayed radiographs post-HSG.

## 2 METHODS

### 2.1 Patients

This cohort study enrolled 78 patients who received HSG examinations between February 2018 and July 2019 in Dalian Women and Children's Medical Group. The enrollment criteria were as follows: (1) aged from 24 to 40 years; (2) female; (3) had spontaneous menstrual cycles; and (4) underwent HSG. The exclusion criteria were as follows: (1) allergic to Ultra-Fluid Lipiodol; (2) documented endocrine disorders such as polycystic ovarian syndrome, diabetes, hyperthyroidism, and hyperprolactinemia; (3) fewer than eight menstrual cycles per year; (4) moderate to severe intrauterine adhesion; (5) obstruction at proximal fallopian tube or effusion at the end of the fallopian tube; (6) colpitis; (7) tuberculosis of the uterus or fallopian tube; (8) during the active phase of the acute or subacute uterus or tubal inflammatory disease; (9) bleeding in the uterus or cervix; (10) amenorrhea but pregnancy not ruled out; (11) severe heart or lung disease; and (12) higher than 37.5°C of body temperature three days before HSG. The Ethics Committee of Dalian Women and Children’s Medical Group approved the study (approval number: 2021020).

### 2.2 Data Collection and HSG Procedures

Patients' clinical characteristics were obtained, including age, sex, menstruation, history of pelvic inflammatory disease, and endometritis. All included patients underwent HSG examination 3-7 days after complete cessation of menstruation. After routine disinfection, the double-lumen balloon catheter was slowly inserted into the cervix and inflated with 2-3 mL of gases. Then, the contrast agent was gradually instilled into the uterine cavity. The injection was continued until the uterine cavity was filled or significant discomfort occurred. During the infusion, the instant films (plain pelvic film, uterine cavity filling film, and fallopian tube spill film) were taken; the balloon was emptied, and the catheter was removed. Then, the delayed films were taken after 2 hours and 24 hours. In addition, adverse events during HSG and after HSG were recorded, including visual analog scale (VAS) pain score, vaginal bleeding, and countercurrent blood flow.

### 2.3 Assessment

Two delayed films (at 2 hours and 24 hours) for each patient were randomized and assigned to two folders with the instant films; the two folders were randomized. Then, the films were read by investigators to assess fallopian tube patency, uterine morphology, and pelvic cavity morphology. The tubal patency was classified as (a) patent and smooth: both fallopian tubes were shown well, and the contrast medium dispersed evenly in the pelvic cavity; (b) patent but short of smooth: the speed of contrast medium passing through the fallopian tube was slightly slowed, and the contrast medium dispersed at the fimbriae of the fallopian tube; (c) patent but not smooth: resistance existed when contrast medium was injected. The speed of the contrast medium passing through the fallopian tube was slowed. A small amount of contrast medium remained in the fallopian tube, and a small or medium amount dispersed in the pelvic cavity; (d) patent but extremely not smooth: the resistance when injecting the contrast medium was high. The speed of the contrast medium passing through the fallopian tube was significantly slowed. A large amount of contrast medium remained in the fallopian tube, and a small amount dispersed in the pelvic cavity and (e) blocked.

### 2.4 Statistics

Statistical analysis was performed using R 4.0.4. The consistency of fallopian tube patency, uterine morphology, and pelvic cavity morphology was evaluated using Gwet’s AC1. Gwet’s AC1 ranged from -1 (no agreement) to 1 (100% agreement). In the analysis, Gwet’s AC1, ranging from 0.61 to 0.80, was considered substantial agreement; Gwet’s AC1, ranging from 0.81 to 1.00, was regarded as almost perfect.

## 3 RESULTS

### 3.1 Clinical Characteristics

The mean age of the 78 enrolled patients was 31.2±4.16 years, and the median (interquartile range (IQR) was 31.0 (29.0-34.0) years, ranging from 24.0 to 40.0 years. Fifty-seven (73.1%) patients had regular menstruation, while the remaining 21 (26.9%) had irregular menstruation. Nine (11.5%) patients had a history of pelvic inflammatory disease, including 8 (10.2%) patients with pelvic fluid and one (1.3%) patient with pelvic adhesiolysis. Thirteen (16.7%) patients had a history of endometritis, including 4 (5.1%) cases with endometrial polyps, 3 (3.8%) cases with adenomyosis, 2 (2.6%) cases with myoma of the uterus, 2 (2.6%) cases with endometrial lesions, 1 (1.3%) case with uterine malformation, and 1 (1.3%) case with thin endometrium. Detailed information is shown in Table **1**.

### 3.2 Consistency of Fallopian Tube Patency and Uterine Morphology

The detailed results of the evaluation 2 and 24 hours post-HSG are shown in Table **S1**. The delayed radiographs taken 2 hours post-HSG indicated substantial agreement compared with those taken 24 hours post-HSG regarding the diagnosis of left and right fallopian tube patency, with a Gwet’s AC1 value of 0.624. In detail, 78 (50.0%), 7 (4.5%), 15 (9.6%), and 4 (2.6%) tubes were diagnosed as patent and smooth, patent but extremely not smooth, blocked, and hydrosalpinx, respectively. In addition, the most common inconsistent finding was that 25 (16.0%) patients were diagnosed as patent but not smooth by the delayed radiographs taken 2 hours post-HSG. At the same time, they were patent and smooth by the delayed radiographs taken 24 hours post-HSG Table **2**. Representative images of the patent and smooth, short of smooth, patent but not smooth, patent but extremely not smooth, and blocked are shown in Fig (**S1A-E**), respectively.

### 3.3 Consistency of Uterine Morphology and Pelvic Cavity Morphology

The delayed radiographs taken 2 hours post-HSG indicated an almost perfect agreement compared with those taken 24 hours post-HSG regarding the diagnosis of uterine morphology, with a Gwet’s AC1 value of 0.943 Table **3**. In detail, 67 (85.9%), 2 (2.6%), and 5 (6.4%) patients were diagnosed with normal, filling defect, and uterine malformation, respectively, by delayed radiographs taken at both 2 hours and 24 hours post-HSG.

In addition, the delayed radiographs taken 2 hours post-HSG also indicated almost perfect agreement with those taken 24 hours post-HSG regarding the assessment of pelvic cavity morphology, with a Gwet’s AC1 value of 0.876. In detail, 61 (78.2%) and 8 (10.3%) patients were diagnosed with a normal pelvic cavity and the adhesion of fallopian tube fimbria, respectively. Moreover, the most common inconsistent finding was that 6 (7.7%) patients were diagnosed with the adhesion of fallopian tube fimbria by delayed radiographs taken 2 hours post-HSG. In contrast, they were diagnosed with a normal pelvic cavity by delayed radiographs taken 24 hours post-HSG Table **4**.

### 3.4 Adverse Events

During HSG, the most common adverse events included pain and countercurrent blood flow. In detail, according to the VAS pain score, 29 (37.2%) and 3 (3.8%) patients experienced mild and moderate pain, respectively; in addition, 3 (3.8%) patients suffered from countercurrent blood flow. After HSG, only 9 (11.5%) patients were exposed to mild pain. Vaginal bleeding did not occur during the HSG period or after HSG Table **5**.

## 4 DISCUSSION

During the HSG process, the contrast medium is injected into the uterus. With the diffusion of the contrast medium through the fallopian tube, delayed radiographs could be taken to reveal the morphology of the entire uterus, fallopian tube, and pelvic cavity [11]. However, the diffusion speeds of different contrast mediums vary from each other. Due to its high liquidity and low viscosity, a water-based contrast medium can diffuse into the pelvic cavity within 15-20 min after its injection. In contrast, traditional oil-based contrast medium needs 24 hours to diffuse into the pelvic cavity due to its inferior liquidity and high viscosity [6, 17]. However, precisely because of the quick diffusion of the water-based contrast medium, the enhancement effect of its diffusion image is unpromising, especially in revealing pelvic adhesions [4]. The oil-based contrast medium shows advantages in revealing the details of the lesion in the uterus, fallopian tube, and pelvic cavity compared with the water-based contrast medium [18, 19]. Nevertheless, with the pace of life speeding up, the restrictions for applying traditional oil-based contrast medium are the too-long waiting time for delayed radiographs. The Ultra-Fluid Lipiodol discloses an intermediate viscosity between the conventional oil contrast medium and the water-based contrast medium [14], which allows a faster speed of intrauterine dispersion and implies a shorter waiting time for taking delayed radiographs. Hence, this study was carried out to evaluate the feasibility of taking a delayed radiograph at 2 hours post-HSG by using Ultra-Fluid Lipiodol.

It is meaningful to shorten the waiting time for taking delayed radiographs. First, the patients did not need to return to the hospital for the 24-hour delayed radiographs; a distinct image could also be obtained for an accurate diagnosis. At the same time, for medical institutions and doctors, it also helps them improve their efficiency and avoid wasting medical resources.

As mentioned above, some researchers have tried to shorten the time for the delayed time, and by using Ultra-Fluid Lipiodol, the study discloses that the diagnostic accuracy is promising [16]. In the present study, delayed radiographs were taken 2 hours post-HSG, revealing a high diagnostic consistency with that taken 24 hours post-HSG regarding fallopian tube patency, uterine morphology, and pelvic cavity morphology, which implied the feasibility of taking delayed radiographs 2 hours post-HSG as an alternative to traditional procedures (taking delayed radiographs 24 hours post-HSG). This could be explained by the Ultra-Fluid Lipiodol having a high iodine concentration (480 mg/mL). In addition, it exhibited approximately 2.5 times the viscosity of the water-based contrast medium but relatively lower viscosity compared with the traditional oil contrast medium, which allowed the Ultra-Fluid Lipiodol to diffuse into the pelvic cavity at a faster speed with a good enhancement effect compared with the conventional oil contrast medium [14].

The safety profile, especially the granuloma and pulmonary embolism, is also of great concern during the HSG procedure with an oil-based contrast medium [20-22]. However, the Ultra-Fluid Lipiodol-based HSG procedure exhibits a good safety profile for diagnosing tubal diseases [23-25]. For instance, a retrospective study showed that the most common adverse events for Ultra-Fluid Lipiodol-based HSG procedures include intravasation, pelvic inflammatory disease, and allergic reactions. At the same time, no pulmonary embolism or death is observed [21]. Another two reviews also confirmed the acceptable safety profile of Ultra-Fluid Lipiodol during the HSG procedure [19, 21]. In line with previous studies, the most common safety profile in the present study included pain and countercurrent blood flow. In addition, most adverse events were mild and manageable. These findings reconfirm the safety of Ultra-Fluid Lipiodol during the HSG procedure.

Some shortcomings in the present study should be noted: (1) patients who underwent HSG at our hospital could choose a water-based contrast medium or Ultra-Fluid Lipiodol. The current study only included patients who underwent HSG with Ultra-Fluid Lipiodol as a contrast medium. Therefore, the sample size was only 78 patients in the present study, which was relatively small; (2) the time-point chosen (2 hours post-HSG procedure) was dependent on the clinicians’ experience; therefore, setting more time points (such as 1 hour, 2 hours, 4 hours) might elevate the generalization of the study findings; (3) the combination of the application of Ultra-Fluid Lipiodol with other methods (such as letting patients walk during the period waiting for the delayed radiographs) might further shorten the time for taking delayed radiographs, which should be determined in future studies; and (4) the rates of HSG-identified blocked and hydrosalpinx were relatively low. A possible reason was that most of the patients who underwent HSG were from the reproductive center of our hospital, and the reasons for infertility of these patients were quite complex, including polycystic ovarian syndrome, premature ovarian failure, male factor, *etc*.

## CONCLUSION

In conclusion, taking delayed radiographs at 2 hours post-HSG using Ultra-Fluid Lipiodol exhibits high accuracy in diagnosing tubal patency and uterine and pelvic cavity morphology, which may be an alternative to the traditional 24-hour scheme. Considering that taking delayed radiographs at 2 hours post-HSG dramatically shortens the time for waiting, it might further elevate the medical experience for patients and avoid the waste of medical resources.

## Figures and Tables

**Table 1:** Clinical characteristics.

Items	Patients (N = 78)
Age (years)	-
Mean±SD	31.2±4.16
Median (IQR)	31.0 (29.0-34.0)
**Range**	24.0-40.0
Menstruation, No. (%)	-
Regular	57 (73.1)
Irregular	21 (26.9)
**History of Pelvic Inflammatory Disease, No. (%)**	-
No	69 (88.5)
Yes	9 (11.5)
Pelvic fluid	8 (10.2)
Pelvic adhesiolysis	1 (1.3)
**History of Endometritis, No. (%)**	-
No	65 (83.3)
Yes	13 (16.7)
Endometrial polyps	4 (5.1)
Adenomyosis	3 (3.8)
Myoma of uterus	2 (2.6)
Endometrial lesions	2 (2.6)
Uterine malformation	1 (1.3)
Thin endometrium	1 (1.3)

**Abbreviations:** SD, standard deviation; IQR, interquartile range.

**Table 2:** Consistency of left and right fallopian tube patency.

Assessment at 2 Hours	Assessment at 24 Hours						Gwet’s AC1
	Patent and Smooth	Patent but Short of Smooth	Patent but not Smooth	Patent but Extremely not Smooth	Blocked	Hydrosalpinx	
Patent and smooth, No. (%)	78 (50.0)	0 (0.0)	0 (0.0)	0 (0.0)	0 (0.0)	1 (0.6)	0.624
Patent but short of smooth, No. (%)	7 (4.5)	0 (0.0)	1 (0.6)	0 (0.0)	0 (0.0)	0 (0.0)	
Patent but not smooth, No. (%)	25 (16.0)	0 (0.0)	0 (0.0)	0 (0.0)	0 (0.0)	0 (0.0)	
Patent but extremely not smooth, No. (%)	4 (2.6)	2 (1.3)	2 (1.3)	7 (4.5)	3 (1.9)	0 (0.0)	
Blocked, No. (%)	1 (0.6)	0 (0.0)	0 (0.0)	1 (0.6)	15 (9.6)	0 (0.0)	
Hydrosalpinx, No. (%)	4 (2.6)	0 (0.0)	1 (0.6)	0 (0.0)	0 (0.0)	4 (2.6)	

**Table 3:** Consistency of uterine morphology.

Assessment at 2 Hours	Assessment at 24 Hours			Gwet’s AC1
	Normal	Filling Defect	Malformation	
Normal	67 (85.9)	0 (0.0)	2 (2.6)	0.943
Filling defect	1 (1.3)	2 (2.6)	0 (0.0)	
Malformation	1 (1.3)	0 (0.0)	5 (6.4)	

**Table 4:** Consistency of pelvic cavity morphology.

Assessment at 2 Hours	Assessment at 24 Hours					Gwet’s AC1
	Normal	Adhesion of Fallopian Tube Fimbria	Local Adhesion of Pelvic Cavity	Extensive Adhesion of Pelvic Cavity	Others	
Normal	61 (78.2)	1 (1.3)	0 (0.0)	0 (0.0)	0 (0.0)	0.876
Adhesion of fallopian tube fimbria	6 (7.7)	8 (10.3)	1 (1.3)	0 (0.0)	0 (0.0)	
Local adhesion of pelvic cavity	1 (1.3)	0 (0.0)	0 (0.0)	0 (0.0)	0 (0.0)	
Extensive adhesion of pelvic cavity	0 (0.0)	0 (0.0)	0 (0.0)	0 (0.0)	0 (0.0)	
Others	0 (0.0)	0 (0.0)	0 (0.0)	0 (0.0)	0 (0.0)	

**Table 5:** Adverse events.

Adverse events	During HSG	After HSG
VAS pain score, No. (%)	-	-
No	46 (59.0)	69 (88.5)
Mild	29 (37.2)	9 (11.5)
Moderate	3 (3.8)	0 (0.0)
Severe	0 (0.0)	0 (0.0)
Vagina bleeding, No. (%)	-	-
No	78 (100.0)	78 (100.0)
Yes	0 (0.0)	0 (0.0)
Countercurrent blood flow, No. (%)	-	-
No	75 (96.2)	-
Yes	3 (3.8)	-

**Abbreviations** HSG, hysterosalpingography; VAS, visual analog scale.

## Data Availability

The data and supportive information are available within the article.

## LIST OF ABBREVIATIONS

HSG: Hysterosalpingography
VAS: Visual Analog Scale
IQR: Interquartile Range

## STANDARDSOF REPORTING

STROBE guidelines were followed.
