# Supplementary material for: Comparison of the Diagnostic Consistency between Delayed Radiographs taken Two Hours and Twenty-four Hours Post Hysterosalpingography using Ultra-Fluid Lipiodol-based Contrast Medium
Source: Curr Med Imaging. 2025 Sep 18;22:e15734056258980. doi: 10.2174/0115734056258980231112082655 (PMC13639912; doi:10.2174/0115734056258980231112082655)
Supplement: Supplementary file 1 [file CMIM-22-E15734056258980_SD1.pdf]

1875-6603/26 *Send Orders for Reprints to reprints@benthamscience.net*

BENTHAM SCIENCE

CrossMark

Current Medical Imaging

Content list available at: https://benthamscience.com/journals/cmir

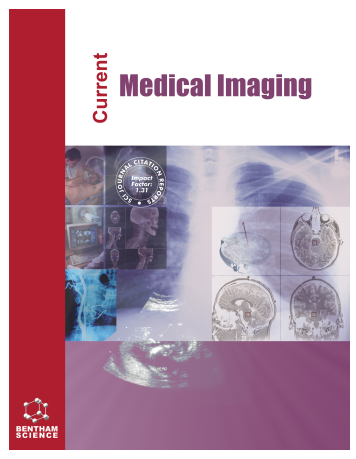

Supplementary Material

Comparison of the Diagnostic Consistency between Delayed Radiographs taken Two Hours and Twenty-four Hours Post Hysterosalpingography using Ultra-Fluid Lipiodol-based Contrast Medium

Yitang Wang[1,*,£]

[1]*Department of Hemangioma and Intervention, Dalian Women and Children's Medical Group, Dalian, China*
[£]*Present Address: Shenyang Medical College Affiliated 242 Hospital First Interventional Department, China*

Table 1. Left and right fallopian tube patency, uterine morphology, and pelvic cavity morphology.

Items	Assessment at 2 hours	Assessment at 24 hours
**Left and right fallopian tubes' patency**	-	-
Patent and smooth, No. (%)	79 (50.6)	119 (76.3)
Patent but short of smooth, No. (%)	8 (5.1)	2 (1.3)
Patent but not smooth, No. (%)	25 (16.0)	4 (2.6)
Patent but extremely not smooth, No. (%)	18 (11.5)	8 (5.1)
Blocked, No. (%)	17 (10.9)	18 (11.5)
Hydrosalpinx, No. (%)	9 (5.8)	5 (3.2)
**Uterine morphology**	-	-
Normal, No. (%)	69 (88.5)	69 (88.5)
Filling defect, No. (%)	3 (3.8)	2 (2.6)
Malformation, No. (%)	6 (7.7)	7 (9.0)
**Pelvic cavity morphology**	-	-
Normal, No. (%)	62 (79.5)	68 (87.2)
Adhesion of fallopian tube fimbria, No. (%)	15 (19.2)	9 (11.5)
Local adhesion of pelvic cavity, No. (%)	1 (1.3)	1 (1.3)
Extensive adhesion of pelvic cavity, No. (%)	0 (0.0)	0 (0.0)
Others, No. (%)	0 (0.0)	0 (0.0)

DOI: 10.2174/0115734056258980231112082655, 2026, *22*, e15734056258980

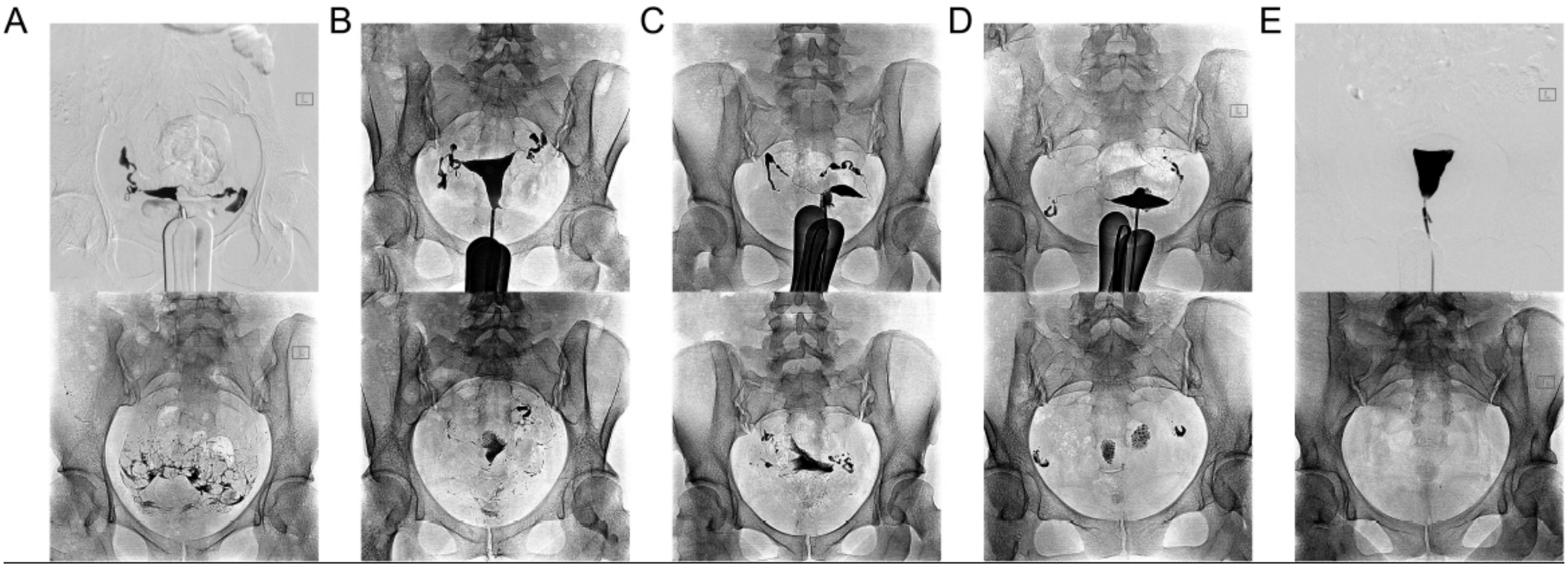

Fig S1. Representative images of tubal patency. Representative images of patent and smooth (**A**), patent but short of smooth (**B**), patent but not smooth (**C**), patent but extremely not smooth (**D**), and blocked (**E**).

© 2026 The Author(s). Published by Bentham Science Publisher.

This is an open access article distributed under the terms of the Creative Commons Attribution 4.0 International Public License (CC-BY 4.0), a copy of which is available at: https://creativecommons.org/licenses/by/4.0/legalcode. This license permits unrestricted use, distribution, and reproduction in any medium, provided the original author and source are credited.

HOW TO CITE:
Wang Y. Comparison of the Diagnostic Consistency between Delayed Radiographs taken Two Hours and Twenty-four Hours Post Hysterosalpingography using Ultra-Fluid Lipiodol-based Contrast Medium. Curr Med Imaging, 2026; 22: e15734056258980. http://dx.doi.org/10.2174/0115734056258980231112082655
